# Endovascular Embolization of Spontaneous Iliopsoas Hematoma: First Experience with Squidperi

**DOI:** 10.1155/2018/4694931

**Published:** 2018-02-27

**Authors:** Pierluca Torcia, Silvia Squarza, Umberto G. Rossi, Paolo Rigamonti, Lorenzo Carlo Pescatori, Giovanni Damiani, Maurizio Cariati

**Affiliations:** ^1^ASST Santi Paolo and Carlo, San Carlo Borromeo Hospital, Department of Diagnostic Science, Radiology and Interventional Radiology Unit, Via Pio II 3, 20153 Milano, Italy; ^2^Department of Diagnostic Imaging, Interventional Radiology Unit, Galliera Hospital, Mura delle Cappuccine 14, 16128 Genova, Italy; ^3^Postgraduation School in Radiodiagnostics, Università degli Studi di Milano, Via Festa del Perdono 7, 20122 Milano, Italy

## Abstract

A 79-year-old man, suffering from atrial fibrillation and on anticoagulation therapy, was admitted at the emergency department of our institution because of a worsening respiratory insufficiency. After a diagnostic work-up, he was found to suffer from pneumonia, and antibiotic therapy was settled. He was kept under observation for his pulmonary conditions but, within a week, he developed a spontaneous iliopsoas hematoma, due to a sudden dysregulation of anticoagulation therapy subsequent to new in-hospital treatments. An endovascular approach was attempted and the bleeding vessels were embolized with a new liquid agent, named Squidperi (Emboflu, Switzerland). Complete exclusion of the diseased vessels was obtained and no complications occurred after the procedure. We conclude that Squidperi can be considered as an option for treatment of spontaneous iliopsoas hematomas.

## 1. Introduction

The incidence of spontaneous iliopsoas hematoma is 0.1% in general population and 0.6% in elderly patients receiving anticoagulant therapies or affected by coagulopathies [1].

As typically it is not considered a life threatening condition, a nonoperative approach should be attempted at first. In fact, a correction of the International Normalized Ratio (INR) can be sufficient to let the bleeding stop. More radical handling (i.e., endovascular or surgical therapies) should be taken into consideration when dealing with continuous and/or consistent bleeding, leading to hemodynamic instability [2].

At present, when an invasive treatment is needed, percutaneous management by selective embolization of the bleeding vessels is considered as gold standard [3].

Herein, we describe a case of spontaneous iliopsoas hematoma treated with Squidperi (Emboflu, Switzerland), a new formulation available for embolization procedures.

## 2. Case Report

A 79-year-old man was admitted at the emergency department of our institution because of a worsening respiratory insufficiency. After a first evaluation he was found to suffer from lobar right pneumonia, so he was hospitalized to receive proper antibiotic treatment and supportive medical care. At the time of admission, he was on oral anticoagulation therapy because of a persistent atrial fibrillation. After a week of treatment, his haemoglobin (Hb) level decreased by 1.5 g/dl (having 10 g/dl at admission). He did not report other symptoms and his blood tests were otherwise consistent with the previous ones obtained during hospitalization. Because of his pulmonary condition, a bronchial artery haemorrhage was suspected, so he underwent urgent Multidetector Computed Tomography (MD-CT).

**Figure 1:**
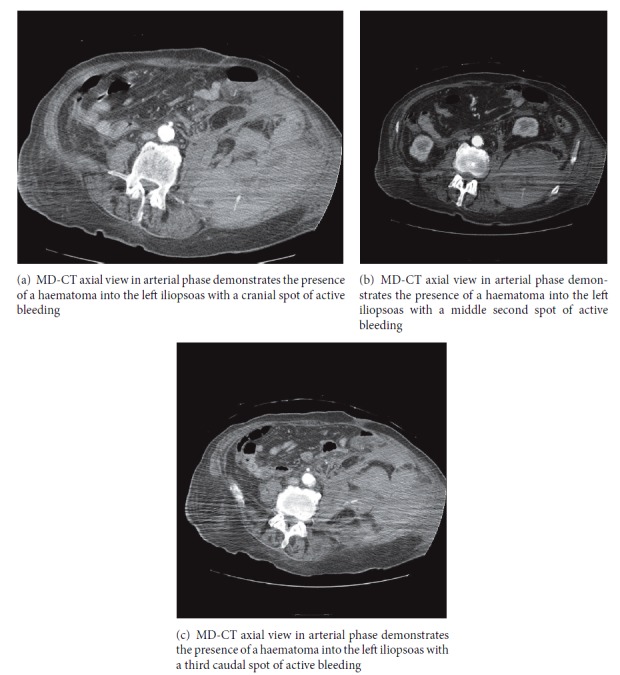


A thoracic-abdominal CT with triphase technique was performed and it demonstrated a left iliopsoas hematoma with three active points of contrast extravasation (Figures 1(a)–1(c)). No active bleeding was detected at pulmonary level.

**Figure 2:**
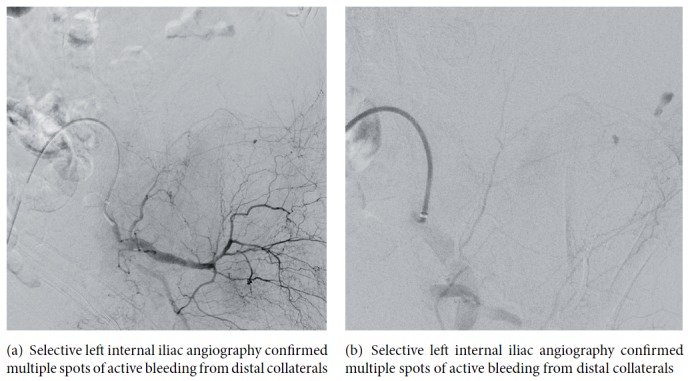


**Figure 3:**
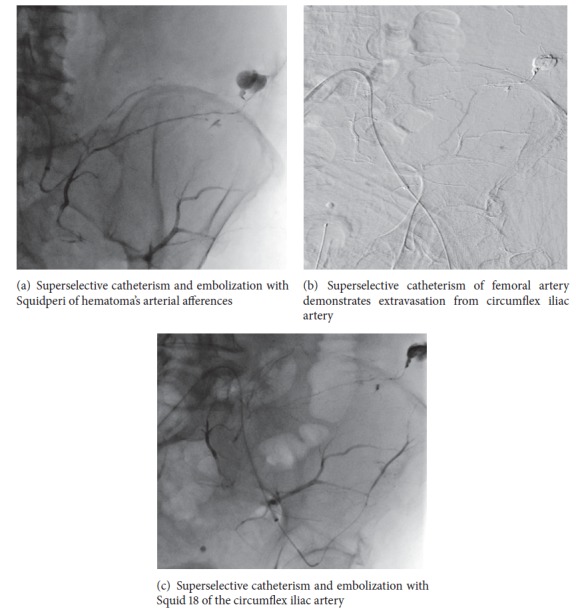


**Figure 4:**
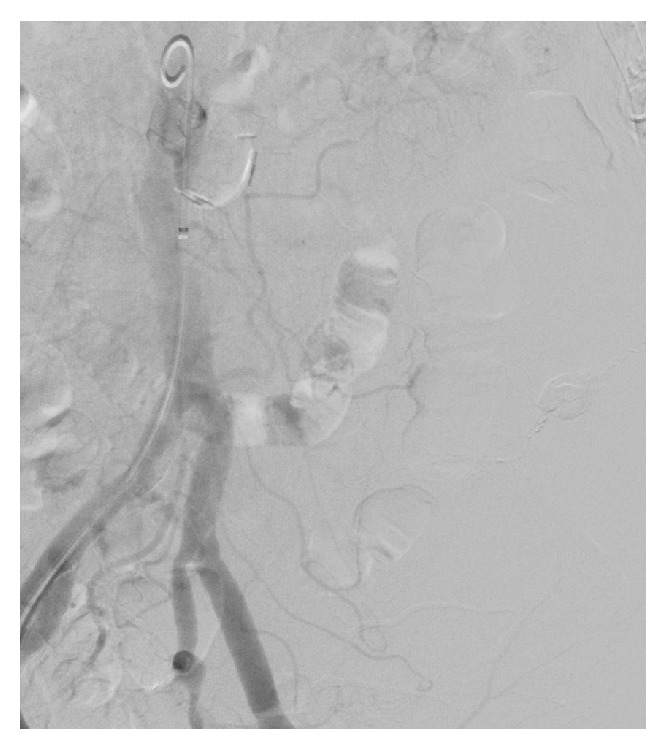
Final angiographic control demonstrates the complete exclusion of hematoma.

After multidisciplinary agreement, the patient underwent urgent Digital Subtraction Angiography (DSA) that confirmed the three points of bleeding described on CT, that is, gluteal artery, iliolumbar artery, and lumbar arteries (Figures 2(a) and 2(b)). DSA also demonstrated a site of active bleeding from circumflex iliac artery (Figure 3(a)). A coaxial superselective catheterization was performed for all these arteries with a Terumo Progreat microcatheter 2.7-Fr (Terumo, Tokyo, Japan) and then all the bleeding spots were embolized using the liquid embolic agent Squidperi 18 (Figures 3(a) and 3(b)). The final DSA control confirmed the complete embolization of all the diseased vessels and absence of active residual bleeding (Figure 4). The postoperative course was uneventful. Patient was discharged 3 days later, after completion of medical therapy for his pneumonia.

## 3. Discussion

Spontaneous iliopsoas hematoma is commonly found in elderly patients on anticoagulant therapies. As perfect compliance to anticoagulation treatments is not easy to obtain, patients' INR can be easily found over the therapeutic range for a number of reasons, and this is the main risk factor for spontaneous bleeding [2]. Likewise, several treatments can react with the anticoagulation drugs, leading to a sudden alteration of INR, like in the case presented.

Anyhow, spontaneous retroperitoneal bleeding is rare and detection can be difficult. In a typical setting, patients may report acute low back pain, but it can be underestimated (both by the patient and the clinician) and referred to spinal neuromuscular problems. However, when the pain is associated with decreasing Hb values and peripheral neurologic symptoms (due to compression by the increasing hematoma), the clinician should suspect a retroperitoneal bleeding [2].

A triphase multislice CT angiography is considered the gold standard for diagnosis, as it allows detection of site and extension of hematoma as well as the evidence of active bleeding presenting as a blush of contrast media in arterial CT phase, that is, an indication for immediate treatment [4].

As stated, different approaches can be proposed when dealing with an iliopsoas hematoma and, at present, there is no general agreement about the best treatment options [3]. In our clinical practice, when a patient is found with a retroperitoneal hematoma but without signs of active bleeding or hemodynamic instability, a medical treatment is firstly proposed. Conversely, in case of blood loss and active arterial bleeding detected by CT scan, endovascular treatment is the first choice. Venous bleeding presents no indications to be treated with endovascular approach and, in case of patient's clinical instability, surgical approach is the only choice of treatment.

Angiography has multiple advantages in this cases as, thanks to high resolution images and to injection of intra-arterial contrast media, it allows confirming the diagnosis obtained with CT scan and searching for other bleeding points that can be missed with other diagnostic modalities. This particular role was demonstrated also in our case. Moreover, once the bleeding vessels are detected, they can be selectively embolized, saving surrounding tissue [5].

Because all of these reasons, surgery should be proposed only after endovascular failure, or when other surgical conditions occur or in case of significant compressive neurological symptoms [6].

After treatment, CT angiography has to be repeated after treatment only in case of unexpected clinical or hemodynamical instability to evaluate the presence of new bleeding; it can also play a role in recognizing complications such as pseudoaneurysms during the follow-up.

To our knowledge, there are no previous reports regarding the embolization of a spontaneous iliopsoas hematoma with Squidperi. It is a nonadhesive liquid embolic agent made with EVOH (ethylene vinyl alcohol) copolymer dissolved in DMSO (dimethylsulfoxide), and suspended in a micronized tantalum powder. First experiences were reported by Akmangit et al. who extensively used Squidperi to treat arteriovenous malformations, arteriovenous fistula, aneurysms, and tumors [7]. Shortly after, Erbahceci Salik et al. reported the use of Squidperi to embolize a pelvic arteriovenous malformation [8]. Recently our group described the successful use of Squid for the management of a spontaneous rectus sheat hematoma [9].

Two forms of the agent are available, with decreasing viscosity: Squidperi 12 and Squidperi 18.

The biggest advantages in using the Squidperi are the high homogeneity of suspension and its stability over time, which allows an easily controlled and prolonged injection. Squidperi presents also a homogenic radiopacity that improves the assessment of vascular architecture

The main limitation of Squidperi is the time needed to prepare the formulation (about 15–20 minutes), mainly in emergency cases. However, if the preparation starts before the superselective cannulation of the bleeding vessels, it can be efficiently delivered without losing precious time.

In conclusion, due to the good intraprocedural result obtained with this embolic agent, it can be considered for treatment of spontaneous iliopsoas hematomas, although further studies are needed to state the outcome of patients on long-term basis.
